# The Role of Volunteers in a Swimming Organization for Persons with Disabilities

**DOI:** 10.3390/healthcare10112149

**Published:** 2022-10-28

**Authors:** Alena Vernerova, Ivana Marova, Frantisek Chmelik

**Affiliations:** 1 Faculty of Physical Culture, Palacký University Olomouc, 779 00 Olomouc, Czech Republic; 2 Faculty of Education, Masaryk University, 601 77 Brno, Czech Republic

**Keywords:** leisure time physical activity, volunteering, swimming, social inclusion, disability, adapted physical activity, respite care

## Abstract

Participation in leisure time physical activity (LTPA) has considerable health-related, psychological, and social benefits. However, the involvement of individuals with disabilities is considerably less than that of their peers without disabilities. A higher rate of participation of individuals with disabilities in LTPA may be achieved by the active involvement of volunteers. This study aimed to describe the importance of volunteer involvement in a swimming organization focused on individuals with disabilities, as perceived by all participants, including swimmers with disabilities, their parents, volunteers, and coaches. The organization uses volunteers as swimming instructors who work individually with swimmers with disabilities. The data were obtained through 11 semi-structured interviews with swimmers with disabilities and their parents, volunteers, and coaches. The interviews were transcribed verbatim and analyzed using a five-step inductive thematic analysis. As a result of the cooperation with the volunteer swimming instructors, swimmers with disabilities felt an improved range of movement, greater independence, and higher self-esteem than before they started using the services of the swimming organization. Consequently, even individuals with severe disabilities can participate in LTPA. Membership to the organization also provided space for the establishment of new social relations, and the instructors described them accepting persons with disabilities as their equals. More importantly, the involvement of volunteers enables organizations to provide respite care for parents.

## 1. Introduction

The full and effective participation of an individual in society includes involvement in leisure time physical activity (LTPA). In addition to the proven health-related benefits, there are significant psychosocial implications.

LTPA has the potential to increase self-esteem and independence in daily life activities for persons with disabilities, leading to an improved quality of life [1]. It also creates a natural space for the socialization of individuals [2] and families with children with disabilities, especially those with severe forms of disability [3]. Physical activity can also lead to improvements in mental health and well-being at any age [4,5]. On the other hand, a lack of movement is a risk factor for declining skills with increasing age [6].

The participation of children with disabilities in leisure time activities may also be beneficial for their parents and other caregivers. Being a caregiver is very mentally demanding and is associated with a high degree of fatigue. Respite care provides caregivers with a short relief period and has the potential to improve their mental health and decrease the extent of long-term fatigue [7]. However, the offer is very limited, and caregivers tend to have concerns about the quality of the service or, by using the service, may have a feeling of failure in their role as a carer [8]. Respite care can also include camps or other types of organized LTPA, which have an added value not only for parents but also for their children with disabilities [9].

A suitable type of physical activity for persons with disabilities, often recommended by physicians, is swimming [10]. In addition to many other health benefits, movement in water allows people with disabilities to be free of the need to use compensatory devices and, to a large extent, the need for assistance [11].

Despite the considerable benefits that have been identified, persons with disabilities participate in LTPA significantly less often than their peers without disabilities [12,13]. This may be influenced by several external barriers such as structural barriers [14,15,16,17], insufficient financing, and low awareness of the possibility of participation [18,19,20,21], as well as internal factors, including, for example, a lack of motivation [22,23], personality factors of the individuals [24,25,26], and the presence of pain [27].

Volunteers represent an important element in increasing the involvement of people with disabilities in LTPA. The involvement of volunteers in LTPA has the potential to increase community-like feelings. Conversely, the absence of volunteers is considered a barrier to the participation in LTPA of persons with disabilities [28]. The quality of volunteer participation involves their feeling of meaningfulness of the perceived benefits [29]. These benefits include the opportunity to improve their organizational and communication skills, acquire new contacts, and learn to work in a team [30]. Student volunteering is perceived as having an added value by society. Thus, it increases the social status of volunteers, thereby increasing their chances of finding jobs and securing admission in universities owing to it being perceived as an asset [31].

Although the involvement of volunteers can be beneficial for all participants, few studies have considered it a factor in facilitating the involvement of persons with disabilities in LTPA. The present study aimed to describe the importance of volunteer involvement in a swimming organization focused on individuals with disabilities as perceived by all participants, including swimmers with disabilities, their parents, volunteers, and coaches.

## 2. Materials and Methods

### 2.1. Research Sample

#### 2.1.1. Settings

This study involved a swimming sports club that focused on individuals with disabilities. This particular sports club was chosen because of organizational factors and the size of the club, which made it able to address enough participants. The swimming club was a nonprofit organization founded in 1995. It focused on leisure time activities, sports, rehabilitation, and swimming lessons for persons with disabilities. In addition to the swimming lessons, the club participates in para-swimming competitions and organizes summer swimming camps. The target group includes persons with disabilities from all age groups. Swimmers were divided according to their skills into groups of competitive, recreational, and rehabilitation swimmers. Recreational and rehabilitation swimmers were individually led by volunteer instructors who were in the pool with them during the swimming lesson. Volunteers and coaches in the organization not only participated as swimming instructors but also provided persons with disabilities assistance or other additional support, especially during swimming camps. The club had few paid staff; therefore, its operation was largely based on volunteers.

#### 2.1.2. Recruitment

The study was conducted in accordance with the Declaration of Helsinki and approved by the Ethics Committee of the Faculty of Physical Culture, Palacký University, Olomouc. Informed consent was obtained from all the participants involved in the study.

For the purposes of the qualitative research, participants from each of the following groups were selected: (a) swimmers, (b) parents, (c) volunteers or coaches (referred to as instructors), and (d) one female participant who was both a swimmer and an instructor. Eleven interviews were conducted (three swimmers, three parents, four instructors, and one swimmer or instructor were interviewed). The interviewees were not parents of the interviewed swimmers. The description of the participants is presented in Table 1. To preserve anonymity, the names of the participants were replaced with pseudonyms.

The sample was established through deliberate sampling. The swimmers were contacted via their coaches. The inclusion criterion was an age of 15 years or older, and the exclusion criterion was an intellectual disability to provide relevant information. The first three swimmers who responded to the call were included in the study. The study sample also comprised parents who accompanied their children to the training sessions. Interviews with these parents were conducted during their children’s swimming sessions and included parents of individuals with severe disability who needed all-day care. We assumed that the impact of the presence of volunteers in the organization on these parents would be more significant than in the case of parents of swimmers with mild or moderate disabilities. The instructors were chosen according to their length of experience and included new instructors with <5 years of experience in the organization, as well as experienced instructors with a history of >5 years, to ensure that various views and experiences were collected.

### 2.2. Procedures and Techniques

#### 2.2.1. Data Collection

Data collection was performed through semi-structured interviews conducted by the first author. The interviews with swimmers and instructors had similarly structured questions and lasted for approximately 30 min. The interviews with parents lasted for approximately 20 min. The interviews were recorded, transcribed verbatim, and anonymized. The semi-structured interviews with all respondents were thematically divided into three parts. The first part was related to the characteristics of the individual, his or her interests, and previous experiences with LTPA. The second part focused specifically on the course of the training sessions, their interactions with their instructor, and the motivation for swimming, whereas the third part focused on the relationships with the organization and other events held by the organization, especially summer camps. The last part also included questions on the subjectively perceived positives and negatives of involvement in leisure organizations. The questions differed slightly for each group to make them relevant according to the role of the respondents within the organization. All responses were based on a retrospective view of the time spent in the organization and their recent feelings.

#### 2.2.2. Data Analysis

The data were analyzed by the first author using a five-step inductive thematic analysis approach [32]. A thematic analysis method provides flexibility and enables the researcher to obtain an in-depth view when analyzing and reporting the themes within the data. In contrast to grounded theory or interpretive phenomenological analysis, this approach does not demand deep theoretical and technological expertise; therefore, it is more suitable for researchers early in their qualitative research careers, a description applicable to the first author [32]. In the first step, the interviews were transcribed verbatim by the first author and read repetitively to immerse herself in the data, search for patterns, and make coding notes. The transcribed interviews were encoded as a second step. Third, the codes were analyzed into subthemes and themes and combined into relevant overarching themes. Fourth, the key themes and subthemes were reviewed to ensure their relevance and entered into the data set [33]. Fifth, the themes were delivered to each group of participants separately (swimmers, instructors, and parents), after which the first author searched for relevant intersections between the groups. As a final part of the data analysis, the themes were reviewed by all the authors to ensure their relevance to the aim of the study.

For clarity, the authors evaluated the frequency of occurrence of the subthemes in each participant’s group (Table 2). “X” illustrates a high frequency of occurrence, which was set as >15 mentions among swimmers and instructors and >10 among parents, “x” illustrates a medium frequency of occurrence, which was set as 10–14 mentions among swimmers and instructors and 5–9 among parents, and “o” illustrates a low frequency of occurrence, which was set as 1–9 mentions among swimmers and instructors and 1–4 among parents.

## 3. Results

Three overarching themes were constructed based on the key themes and subthemes that emerged from the data analysis: (1) “Personal enrichment” described the individual benefits for all the participants, (2) “Social interactions” illustrated the social impact of the involvement in the organization, and (3) “Family support” represented the benefits for all families with children with disabilities. All themes were based on the cooperation of swimmers with the volunteers in the organization. Table 2 presents each subtheme reflecting the frequency of mentions of the specific groups of participants in the interviews (see Data Analysis). There are three tables that present the significant expressions for each subtheme according to the groups of participants that supported the research outcomes. Table 3, Table 4 and Table 5 present quotes from the swimmers, instructors, and parents, respectively.

### 3.1. Personal Enrichment

The overarching theme of personal enrichment comprised three key themes: health and well-being, self-esteem, and new possibilities.

Health and well-being: Swimmers often reported the rehabilitation aspect of swimming with an instructor. They experienced an improvement in their range of movement. Thanks to swimming, Elena reported experiencing not only a larger range of movement but also a greater awareness of her body (see Table 3).

Instructors mentioned that, thanks to the individual lessons, they could help swimmers stretch their stiff muscles during the training session and help them reach their body potential. They described how they liked to observe the improvement in the movement of the swimmers throughout the swimming lessons (see Table 4).

The same effect was observed by the parents in their children with disabilities; they reported mostly a greater range of motion, which they attributed to swimming, as mentioned by Hynek, a father of a 24-year-old man with a severe type of cerebral palsy (see Table 5).

Moreover, the swimmers described how water provided them with possibilities of movements that they were unable to perform outside water (for example, walking in water and a more effective relaxation of spasticity).

In addition to the physiological benefits, swimming also produces a psychological effect, as demonstrated by the reports from swimmers, such as Gustav, where most described the freedom they felt during swimming sessions (see Table 3). Parents appreciated that swimming offered their children a great opportunity to move, and in their opinion, it compensated for their children having to sit all day in a wheelchair.

Based on descriptions from the swimmers, the feeling of safety while swimming with an individual instructor was an important factor, which helped the swimmers to relax and fully enjoy the swimming sessions (see Table 3). The instructors often mentioned that they were not only focused on the swimming progress but mostly on the positive experiences of the swimmers and their own enjoyment in assisting the swimmers (see Table 4). They stressed the individual approaches and communication during this process. The presence of individual instructors was also an important factor for the parents, who reported that having an instructor of the opposite sex provided an additional unplanned motivation to the swimmers (see Table 5).

Self-esteem: Both the swimmers and instructors perceived a feeling of higher self-esteem due to their participation in the swimming organization. It was not linked only to the swimming lessons but also to the other events organized by the sports club. For example, Elena described how she succeeded in traveling all by herself to get to the club’s event (Table 3). Thanks to the cooperation between the swimmers and instructors, both groups described how they had to overcome various challenges, which made them feel more confident in their abilities. In particular, the instructors described that they had to overcome their own fears during the training sessions, as stated by Claudia (see Table 4). Involvement in the organization brought a sense of meaningfulness to both instructors and swimmers. At the same time, they shared a feeling of fulfillment when swimmers made progress. According to both swimmers and instructors, this feeling of meaningfulness was important for strengthening their self-esteem (see Table 3 and Table 4).

New possibilities arose among swimmers consequent to their becoming more independent, thanks to the trust, safe space, and support that they received from the instructors. Although swimmers with severe disabilities would not be able to participate without an instructor, some swimmers gradually became independent of their instructors and managed to swim on their own. This kind of progress was described by Iva, a parent of a 25-year-old blind woman with cerebral palsy (see Table 5). The feeling of independence described by the swimmers, was not only at the physical level but also at the psychological level when they could experience new life situations unaccompanied by parents, especially during the camps. Fiona especially enjoyed that they were not taken care of as children, which was quite common in other organizations based on her experience (see Table 3). The instructors mentioned having a different perspective on life owing to their involvement in the organization, and furthermore, this played a role in the instructors’ choices for their future jobs. As Anna described, it helped her to consider her future path (see Table 4). Similarly, some swimmers became swimming coaches and, thus, served as models for other swimmers. A good example was Denisa, who used to be a swimmer at the international championship level but now participates in the organization as an instructor (see Table 4).

### 3.2. Social Interactions

The overarching theme of social interactions comprised two key themes: community and social inclusion.

Community: The organization had a friendly atmosphere and created a community where everyone felt welcomed. Therefore, it was easy to establish a relationship between the swimmers and instructors. Good relationships played a major role in keeping participants in the organization. Parents often mentioned how their children felt safe and welcomed in the community. Jan, the father of a 37-year-old woman who experienced traumatic brain damage, described how good friendships was one of the main reasons why his daughter attended the swimming sessions. Another parent, Iva, perceived that her daughter felt safe in the community (see Table 5). Swimmers had the opportunity to establish relationships with individuals with similar disabilities, as well as with people without disabilities. This was further confirmed by the fact that the swimmers and instructors often met outside training sessions and became friends, as Anna described (see Table 4). Participants from all groups (swimmers, instructors, and parents) often talked about the organization as if it were a family.

Social inclusion: Participants with disabilities described feeling equal to their instructors. In the words of Fiona, a swimmer, “swimmers and instructors are at the same level” (see Table 3). In addition, the instructors gained experience with people with disabilities. As a result, they accepted them as a normal part of society, and they overcame prejudices. This phenomenon was expressed by Claudia, an instructor, who said that it changed her view of people with disabilities (see Table 4).

### 3.3. Family Support

The last overarching theme was family support, and the main theme that emerged was respite care and its benefits for both parents and swimmers.

The swimmers benefited mainly from changing their environment and becoming independent of their parents, with whom they spend much more time than their peers without disabilities. In this way, they had the opportunity to get to know themselves in different social roles other than as a daughter or son. Fiona, a swimmer, described the swimming camp as an “antieducational camp”, where she could be by herself without parents and personal assistants (see Table 3).

For parents, the swimming organization provided a substitute for respite care. During training sessions led by instructors, parents had time to rest from their caring responsibilities. An even stronger effect was described in talking about summer swimming camps where the volunteers worked as both instructors and personal assistants to the swimmers. These camps were beneficial for swimmers and their parents.

The parents appreciated that their children were taken care of by the instructors and that they could rest for a week. As Jan stated, he could go on a getaway trip with his wife to gain some energy for the year (see Table 5). The same benefits were perceived by the instructors, as Denisa realized even half an hour (the time of the swimming session) of the parents being apart from their children with disabilities proved to be very refreshing for both groups (see Table 4).

## 4. Discussion

All respondents agreed on the benefits stemming from the cooperation between the swimmers and instructors in the swimming organization. The participants described how they felt a greater range of movement and that they could perform movement patterns that they were unable to perform by themselves on the ground, thanks to cooperation with the instructors. The aquatic environment made the swimmers feel free and independent. Although the instructors attempted to lead swimmers toward the greatest possible degree of independence, simultaneously, they provided a safe environment that had the potential to increase the effect of this development. The development of independence and self-reliance in persons with disabilities can lead to an improved quality of life [1].

Another positive aspect was the sense of meaningfulness perceived by participants in the organization. The individuals included in the research described a feeling of freedom, meaningful fulfilment of free time, and consequently, increased self-esteem. Owing to sports, individuals with disabilities often feel pride, meaningfulness, and freedom [34]. In the present study, these feelings were described by both the swimmers and volunteers. Previous studies have suggested a connection between volunteering and well-being, higher self-esteem, and a greater sense of belonging [35].

The positive climate in the organization was one of the main reasons why participants stayed. The swimmers and instructors often became friends, even outside the organization. Similar results were suggested by a study on participation in a wheelchair basketball organization, where participants felt welcomed and appreciated among their peers without disabilities [36]. This is a good step toward the inclusion of persons with disabilities in mainstream society. The direct experience of instructors with individuals with disabilities was subjectively correlated with better attitudes towards this group, which is consistent with the results from other studies [37,38,39]. The finding that the connection between individuals with and without disabilities supported a sense of belonging, respect, and acceptance, especially among persons without disabilities [28], was also supported by the results of the present study.

Leisure time organizations and sports clubs also play an important role in respite care. Families often feel a lack of opportunities for leisure, recreation, and social activities for their children with disabilities. At the same time, these activities give parents a necessary respite from all-day care [40]. Camps, which, in the present study, were summer swimming camps, were considered particularly beneficial by parents, as they could go on a holiday knowing that their child would be taken care of. The children are cared for by the instructors, without whom these camps would not take place. The findings of the present study cannot be generalized; however, other studies have confirmed that camp-like events had a positive impact on caregivers’ mental health [9]. At the same time, it allowed individuals with disabilities to change their environment and provided an opportunity for recreation and personal growth [41]. The results of the present study were similar.

These results suggested several benefits of volunteer involvement in providing LTPA to individuals with disabilities. Despite the small sample size, this study clearly showed some positive patterns. It appeared that the involvement of volunteers contributed to improving the conditions for individuals with disabilities and their families. Therefore, greater attention should be paid to LTPA as a form of respite care, as this is an inspiring idea that has so far not been optimally used in the Czech Republic and in many other countries.

In contrast to previously published results [8], the parents in our study did not report any concerns about the quality of the services provided by the organization or that they felt unsuccessful in their role as carers when using the service. Future studies should therefore also address parents’ possible negative perceptions about volunteers in leisure time organizations and sports clubs.

### Limitations and Strengths

The first author had her own experience as an instructor in the organization. Despite her efforts to achieve the highest degree of objectivity, she realized that her experience could have been reflected in the interpretation of the results of the study. The study showed positive patterns; however due to the small sample size and specificity of the deliberately chosen swimming organization, the results cannot be generalized. It is unclear to what extent the results would be transferable to different sports organizations for people with disabilities. Therefore, future studies in different sports settings are encouraged to enable an investigation of other sports, with all the practical implications. Another limitation of this study was the short duration of the interviews conducted. A strength of the study was the comprehensive approach to the issue, which was analyzed from the perspectives of all the actors in the organization.

## 5. Conclusions

Owing to the cooperation between volunteers and coaches in the role of swimming instructors and swimmers with disabilities, the swimmers described a subjectively perceived improvement in their range of movement, increased independence, and development of self-esteem. Through their activities, the instructors helped individuals with disabilities participate in the organization. Moreover, involvement in the organization brought about new social relationships that continued to exist outside the organization. Thanks to these interactions, the instructors reported that it helped them accept people with disabilities as a normal part of society. The swimmers and volunteers described their involvement in the organization as a meaningful way of spending free time, which, according to them, led to a feeling of higher self-esteem. The parents appreciated all of the above benefits, especially that their child felt welcome and safe in the organization, and they valued that their children were taken care of during the swimming lessons and camps, which provided them time to rest from their carer responsibilities.

## Figures and Tables

**Table 1:** Descriptions of the participants.

		Swimmers (n = 4) *	Instructors (n = 5) *	Parents (n = 3)
Age (years), (M ± SD)		21 ± 2.6	24.8 ± 6.2	58.7 ± 5.9
Sex (*n*)	Male	1	2	2
	Female	3	3	1
Occupation (*n*)	Student	4	3	-
	Employed	-	2	2
	Unemployed	-	-	-
	Retired	-	-	1
Diagnosis (*n*)	Cerebral palsy	3	1	-
	Muscular dystrophy	1	-	-
Type of mobility device used	None	2	5	3
(*n*)	Manual wheelchair	1	-	-
	Power wheelchair	1	-	-
Participation (years), (M ± SD)		12.5 ± 8.2	5.8 ± 5.1	13.7 ± 4.6

* One participant provided two interviews, one in the role of a swimmer and another as an instructor; thus, her data was included in both groups. M, mean; SD, standard deviation.

**Table 2:** Key themes and subthemes mentioned by the participants.

Overarching Themes	Key Themes	Subthemes	Participants		
			Swimmers	Instructors	Parents
Personal enrichment	Health and well-being	Movement development	X ^a^	x	x
		Freedom of movement	x ^b^	o ^c^	o
		Enjoyment	X	X	X
	Self-esteem	Empowerment	x	X	x
		Surpassing oneself	o	x	o
		Meaningful time spending	x	X	o
		Swimming progress	o	x	x
	New possibilities	New experience	o	o	x
		Future work direction	o	o	
		Independence	o	o	x
Social interactions	Community	New friendships	X	X	X
		Feeling of belonging	X	X	X
		Safe space	X	x	x
	Social inclusion	Acceptance of disability	o	X	
		Mutual trust	x	x	o
Family support	Respite care	Change of scenery	o	o	o
		Time to rest	o	o	o

^a^ X indicates a high frequency of mentions of a particular subtheme of each participant’s group. ^b^ x indicates a medium frequency of mentions of a particular subtheme of each participant’s group. ^c^ o indicates a low frequency of mentions of a particular subtheme of each participant’s group.

**Table 3:** Swimmers’ quotes according to the mentioned subthemes.

Key Themes	Sub-Themes	Quotes
Health and well-being	Movement development	“It has had a great impact on my daily life. When I move my body, I am aware of which muscle I am using at the moment. It also shows in my dancing skills. Even my coaches tell me I have a bigger range of movement. It has helped me to be more aware of my body and its possibilities.”—Elena
	Freedom of movement	“I enjoy the movement in the water itself, we do what we want. What we can do best. I am not sure how to express it, but it gives us a feeling of freedom.”—Gustav
	Enjoyment	“I enjoy the movement in the water itself, we do what we want. What we can do best. I am not sure how to express it, but it gives us a feeling of freedom.”—Gustav
Self-esteem	Empowerment	“Once there was an event at the end of the season and there was no one who would go with me. So I was supposed to go all by myself by bus and I never go alone by bus because I am too scared. We agreed that someone would pick me up at the bus station but they forgot about me. After all, I got there by myself and felt so proud, because I surpassed myself and stepped up from my comfort zone.” —Elena
	Meaningful time spending	“It is my free time and thanks to swimming, I feel more self-confident because I know I am spending my free time in a meaningful way.—Elena
	Swimming progress	“The first time they threw me into the water, I could barely hold myself on the pool edge when I wanted to talk. So I am able to see the progress.”—Fiona
New possibilities	New experience	“Sometimes it leads to some completely different opportunities. For example, they were filming some documentary, TV show, or something like that. And they wanted to film something about our organization, about the cooperation of swimmers with instructors. They wanted someone who is physically impaired and talkative, so they chose me (laugh).”—Fiona
	Future work direction	“I found out I do not like when people try to motivate me, and that I would rather motivate others. So I became an instructor.”—Denisa, swimmer/instructor
	Independence	“I found out I do not like when people try to motivate me, and that I would rather motivate others. So I became an instructor.”—Denisa, swimmer/instructor
Community	New friendships	“I like the competitions a lot. We are meeting there with our friends from different sport clubs. One could say it is more of a social event than a sports event.”—Gustav
	Feeling of belonging	“It seems to me that everybody is talking to everybody, and the swimmers and instructors are at the same level. That is what I like the most. Sometimes when I see different camps, the program is so childish, like we were babies! So, I like this approach way better.”—Fiona
	Safe space	“I like, that every time we come to the training session you cannot see any person who works with us or whom you meet there that would not like to be there.”—Gustav
Social inclusion	Mutual trust	“When I am at the training session, I know that someone would jump for me into the water if anything happens. At the very moment, when someone appears in front of me and we are swimming together, I feel more secure. I can relax and enjoy it more.”—Elena
Respite care	Change of scenery	“I say it is a bit of an anti-educational camp (laughing). Because we get away from our parents and personal assistants and simply spend time with friends.”—Fiona
	Time to rest	“Because when we are there for the whole week [camp] we simply live there and we get used to going to the restaurants and bakeries, everywhere we want to. I always feel like I am on vacation.”—Fiona

**Table 4:** Instructors’ quotes according to the mentioned subthemes.

Key Themes	Sub-Themes	Quotes
Health and well-being	Movement development	“…so when you are focusing on the right technique when you stretch them in the water when you are forcing the swimmers to prolong the movements, you can help them even from the physiological viewpoint. You can help them to both stretch the muscles and relax the joints.”—Denisa
	Enjoyment	“I just want the swimmer to enjoy the lesson and I also want to enjoy it myself. For me, it is a relaxation. I jump into the water to chill. It also gives me different thoughts.”—Claudie
Self-esteem	Empowerment	“So I tell him: ´Just try to do it two times by yourself, if anything goes wrong, I am here to catch you.´ That’s how I lead them to be more independent.”—Boris
	Surpassing oneself	“Of course, I was scared during the first years of my volunteering. Well, I guess I am still a bit scared. Especially, because I am a technician I don´t know the diagnoses, and I didn´t know what to expect from the swimmers. After some time I discovered that they are actually grateful that I can properly stretch their muscles.”—Claudie
	Meaningful time spending	“I feel like I am doing something meaningful with a part of my free time.”—Boris
	Swimming progress	“Of course, I feel happy when I see that the swimmers are improving. That is probably the thing that fulfills me the most.”—Anna
New possibilities	New experience	“Once I was with her [swimmer] in the Netherlands because she had a boyfriend there at the time. And he wanted her to visit him and her mum felt awkward about the idea of going with her. So I gladly accompanied her.”—Claudie
	Future work direction	I started to volunteer in this organization. It opened my eyes and I realized this could be the right path for me. I still think this could be my future work direction.”—Anna
	Independence	“It is important not to do things instead of them and to trust them, that they can do it by themselves. It is also important to overcome the inner need of helping them and just verbally motivate them to try to do it by themselves.”—Tomas
Community	New friendships	“I feel more like a friend than an instructor to the swimmers. After one and a half year of volunteering in the organization I found many friends among the swimmers. With some of them, we also meet outside the swimming sessions and camps and it just makes me happy that they are more than just the swimmers for me.”—Anna
	Feeling of belonging	“The members of the organization are more open than the mainstream society. They try to understand one another and be kind to each other. (…) They always try to find ways to make things possible than looking for problems. They try to overcome the obstacles together. It is easy to be accepted.”—Tomas
	Safe space	“I have never met a bad person in the organization.” —Anna
Social inclusion	Acceptance of disability	“When I see a person with a disability, I don’t close my eyes anymore, I see it as something normal. I found out that they are normal people, and only some of them carry a chair with them. “– Claudia
	Mutual trust	“No foam board can replace a person. Foam boards can be very unstable. In our training, there are always hands of the instructor so the swimmers can lean on them. You cannot even imagine how huge the difference is. A person gives the swimmer real support.”—Denisa
Respite care	Change of scenery	“Parents get time to rest and to go for a trip and the swimmers can have a beer or French fries without any remorse.”—Claudie
	Time to rest	“The most important thing for both the parents and swimmers is that they are apart for half an hour.”—Denisa

**Table 5:** Parent’s quotes according to the mentioned subthemes.

Key Themes	Sub-Themes	Quotes
Health and well-being	Movement development	“…and we see that the range of movement in the shoulder joint is perfect now. His right arm movement was much worse. Or should I say it was much worse than the left arm. And thanks to swimming his movements of both arms are now almost identical.”—Hynek
	Enjoyment	“He is at the age when he likes girls, naturally. So when he gets to train with a beautiful female instructor, oh how hard he tries!” (laugh)—Hynek
Self-esteem	Empowerment	“I just wanted him to learn how to swim. And I watched their approach towards the swimmers. The instructors are often very good swimmers so they give it their all. And it is visible in the results of the children from the organization. How far they have got! Because some of them were competing in European championships and that is incredible. Also, thanks to the volunteers.”—Hynek
	Swimming progress	“I have never imagined that my son would be able to swim and now he can swim a 200 m backstroke, 200 m at once! That is amazing!”—Hynek
New possibilities	New experience	“That they are taken care of for the whole week in a different environment and she is always full of new experiences and then the whole year draws from it.”—Iva
	Independence	“The swimming progress is great. At first, she was swimming only with assistance, and now we throw her into the water and she can swim by herself.”—Iva
Community	New friendships	“She usually swims with the same instructor and she likes her and the instructor likes my daughter. So this beautiful friendship was created and I think she attends the training mostly because of the instructor.”—Jan
	Feeling of belonging	“Once my nephew drove me and my daughter to the swimming camp and when he saw the warm-hearted welcome and the atmosphere he was amazed. Because he was a person who did not know anything about the organization. He saw it and he told me: ´wow, that is amazing!´”—Jan
	Safe space	“Well if I just mention the fact she always talks about the community where she feels very safe, and when there is a competition ahead, she is always very excited. She just loves the people there. She does not have many friends, which is sad. But she feels very safe among the swimmers and she just loves the whole community.”—Iva
Social inclusion	Mutual trust	“I appreciate that they give all their energy to the swimmers and that the instructors make friendly connections with every single one of them and that they help the swimmers a lot.”—Jan
Respite care	Change of scenery	“I think that, even though she is sometimes just floundering in the water, at least we leave the house once a week, and in a way, she looks forward to swimming, even though she is not achieving any real results.”—Jan
	Time to rest	“I always find a sightseeing tour on the internet, and we set off with my wife for a week. And that makes us satisfied for the rest of the year.”—Jan

## Data Availability

The data presented in this study are available upon request from the authors.
